# Factors associated with social support in child-rearing among mothers in post-disaster communities

**DOI:** 10.1186/s12199-018-0747-7

**Published:** 2018-11-07

**Authors:** Mika Nishihara, Yasuhide Nakamura, Toru Fuchimukai, Mayumi Ohnishi

**Affiliations:** 1 Graduate School of Biomedical Sciences, Nagasaki University, 1-7-1 Sakamoto, Nagasaki, 852-8520 Japan; 2 School of Nursing and Rehabilitation, Konan Women’s University, 6-2-23 Morikita-machi, Higashinada-ku, Kobe, 658-0001 Japan; 3 Iwate Prefectural Ofunato Hospital, 10-1 Yamamagoe, Ofunato-cho, Ofunato, 022-8512 Japan

**Keywords:** Post-disaster community, Social support, Factor analysis, Mothers in child-rearing, Great East Japan Earthquake and Tsunami

## Abstract

**Background:**

Natural disasters have long-term negative impacts on the health and socioenvironmental conditions of a population, affecting the physical environment as well as the relationships within the community, including social networks. Mothers in post-disaster communities may have difficulty receiving social support not only from family members and relatives but also from members of their community, such as people in their neighborhoods. This study focused on mothers with infants and preschool-aged children in post-disaster communities. The associations of social support with sociodemographic characteristics and socioenvironmental conditions related to child-rearing among mothers in post-disaster communities were assessed.

**Methods:**

An anonymous self-administered questionnaire survey was conducted in October 2015 in 988 households in areas affected by the Great East Japan Earthquake and Tsunami. The data collected on sociodemographic and socioenvironmental characteristics included the presence of pre-disaster acquaintances in the neighborhood and social support for child-rearing. The associations of sociodemographic and socioenvironmental characteristics with social support were examined.

**Results:**

We analyzed 215 completed questionnaires from mothers living in different houses from those they lived in before the disaster to reflect continuous relationships with people from the pre-disaster communities. Social support was significantly associated with infant sex, extended family, support obtained from relatives not living together, pre-disaster acquaintances, use of child support resources, and no perceived difficulties in child-rearing. In addition, the presence of pre-disaster acquaintances was associated with categories of mental/physical place of comfort and child-rearing support, with adjusted odds ratios of 1.88 (95% CI 1.03–3.44) and 2.84 (95% CI 1.46–5.52) compared with mothers who did not have any pre-disaster acquaintances.

**Conclusions:**

Factors associated with the obtainment of social support in child-rearing among mothers in post-disaster communities were attributed not only to mothers themselves and family members but also to socioenvironmental factors such as the presence of pre-disaster acquaintances. The presence of pre-disaster acquaintances promoted rich social support in child-rearing in post-disaster communities. When reconstructing a community following changes in residence location after a disaster, the pre-disaster relationships among the community dwellers should be considered from the viewpoint of child-rearing support.

**Electronic supplementary material:**

The online version of this article (10.1186/s12199-018-0747-7) contains supplementary material, which is available to authorized users.

## Background

The Great East Japan Earthquake and Tsunami, which struck the northeastern Japanese region of Tohoku with a magnitude of 9.0 on 11 March 2011, has been described as one of the worst natural disasters in Japanese history. According to the Ministry of Internal Affairs and Communications, 19,575 people lost their lives, 2,577 went missing, and 1,146,371 homes were destroyed or damaged in the disaster [1]. Natural disasters have a large long-term negative impact on the health and socioenvironmental conditions of a population, affecting not only infrastructure but also community-based resources, including social networks [2–6].

Social networks are important for physical and mental health, even under normal conditions in general populations and communities [7, 8]. Social support and good social relationships have an important positive contribution to health, and social support helps to provide emotional and practical resources to people within the community [9, 10].

Studies after Hurricane Katrina in the USA indicated that social support was one of the factors most strongly related to self-rated health [11]. In addition, several previous studies indicated associations of disasters with mental health issues, such as posttraumatic stress disorder, as well as with social support [4, 12–14]. Thus, natural disasters affect not only the physical environment but also the relationships within the community, including social support.

The Great East Japan Earthquake and Tsunami has affected the physical and socioenvironmental conditions of the local communities, including community-based social relationships, workplaces, and child-rearing [15, 16]. A previous study regarding natural disasters indicated that families are extremely important support systems and constitute the most important unit for post-disaster intervention efforts, especially families with children [5]. The environmental situation in post-disaster communities can have an influence on the mental and physical health status of mothers, as well as child-rearing conditions, which could affect their children’s growth and development. A previous study indicated changes in the social and physical environment for mothers and their children after the 1995 Kobe earthquake in Japan due to both direct and indirect effects, and both affected their physical and psychological status [17]. Another study indicated that community support reduced depressive symptoms among mothers following the disaster [18]. These reports suggest that the social and physical environment can influence the mother’s and children’s health, and support from the community is helpful for mothers in raising their infants and preschool-aged children. Additionally, studies on the general population in Japan demonstrated that methods for improving the community environment for mothers were needed to enhance child-rearing support in communities [19, 20], as well as that social support is an important factor in post-disaster communities. However, mothers may have difficulty receiving social support following socioenvironmental changes by disaster, not only from family member and relatives but also from members of their community, such as people in their neighborhoods.

It is suggested that a post-disaster community may exhibit particular characteristics that are influenced by disasters. Therefore, understanding the particular characteristics and current situation of child-rearing for mothers could contribute to discussions and implementation of child-rearing support in post-disaster communities, as well as disaster preparedness in the future.

This study focused on mothers with infants and preschool-aged children in post-disaster communities. We assessed the association of social support with sociodemographic characteristics and socioenvironmental conditions related to child-rearing among mothers living in a house different from that before the disaster as a representation of the population affected by socioenvironmental change. In general, it is important to understand the situation of mothers related to child-rearing and to clarify the strength of mothers provided by social support by taking community characteristics into consideration.

## Methods

**Fig. 1:**
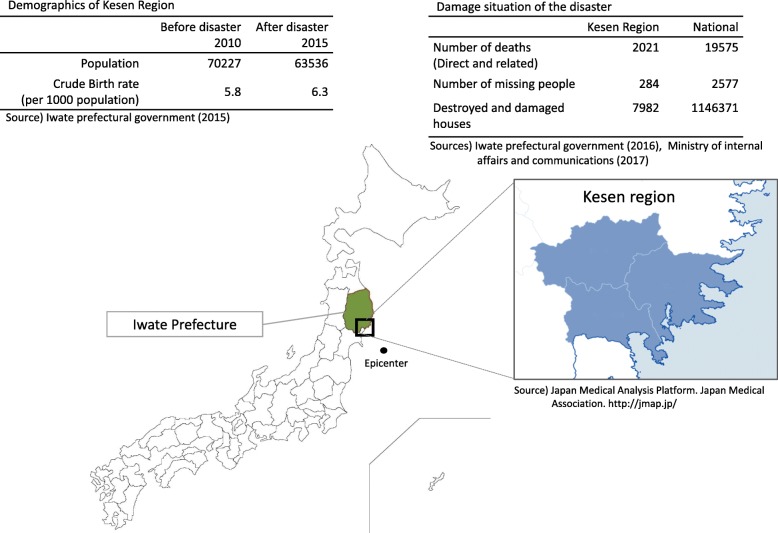
Information of Kesen region

This cross-sectional design study was conducted in the Kesen region, which includes three municipalities in a coastal area of Iwate Prefecture that were heavily damaged in the Great East Japan Earthquake and Tsunami (Fig. 1). The target population was mothers with infants ranging in age from 6 to 42 months old. Information regarding households with infants in this age group was obtained from residence registries from each municipality office in the Kesen region in October 2015. A total of 988 households with infants aged 6–42 months old in this region were invited to participate in this study. An anonymous self-administered questionnaire was mailed to the potential participants and returned directly to the researchers after completion through the postal mailing system. Data were collected on sociodemographic characteristics (infant sex and age (in months), age group of the primary caregiver, household members, employment status of the primary caregiver [not employed, irregular employment, or regular employment], type of housing [independent or non-independent, including multi-family apartments and public temporary housing]), socioenvironmental characteristics (housing change from before the disaster, acquaintances in the neighborhood from before the disaster, support from relatives not living together, and use of child support resources such as a nursery, regional child support center, and mothers’ self-help group), social support for child-rearing, and feelings of difficulty regarding child-rearing. Participants living in a house different from that before the disaster were asked about the presence of acquaintances in the neighborhood from before the disaster (hereafter, “pre-disaster acquaintances”) to reflect continuous relationships with people in the pre-disaster communities. For the purpose of this study, “acquaintances in the neighborhood” included close friends as well as casual acquaintances. The self-reported nature of the response to this question therefore reflected the subjective feelings of the participants about their relationships with individuals in the neighborhood from before the disaster. The responses to this question were classified into four categories (very much, much, little, or not at all) and divided into two groups: “not at all” was categorized as “none,” while the other responses were classified as “yes.”

The questionnaire items developed by Teshima and Haraguchi [21, 22] were used to elicit responses regarding social support for child-rearing and consisted of three categories of social support for child-rearing in the Japanese context: mental support, mental/physical place of comfort, and child-rearing support. The phrasing of some questionnaire items was modified, and a few questions were excluded from this study to adjust for the situation in post-disaster communities. For example, the word “family” was used instead of “couple [husband and wife]” because the proportion of widows was thought to be high due to the disaster and some mothers may have lost a spouse. Additionally, some questions excluded from this study were, for example, asking about the accessibility of nearby parks because there was a lack of places for children to play due to construction work in the entire communities. Finally, 16 of 18 questionnaire items for social support were applied in this study (the questionnaire items are shown in Additional file 1). The definitions of the three categories of social support in this study were based on the results of a factor analysis reported by Teshima and Haraguchi and another study that used the same questionnaire items [21–23] and also took the situation of post-disaster communities into consideration. Mental support referred to the mental stability of the mother for child-rearing through family support. A mental/physical place of comfort was defined as a place outside the home where the mother could talk about child-rearing and comfortably allow the children to play. Child-rearing support was defined as professional and/or friendly give-and-take support in child-rearing. Responses to each question were the following: strongly applicable, moderately applicable, less applicable, and not applicable at all. The responses regarding social support in child-rearing were used to assess the grade of social support by converting them to a score of 1 to 4 for each question, and they were used to define three categories of social support: mental support (score based on five questions [range 5–20]), mental/physical place of comfort (score based on five questions [range 5–20]), and child-rearing support (score based on six question items [range 6–24]). Finally, each score was assigned to one of two groups based on the median: scores greater than or equal to the median were categorized as “many,” while those less than the median was classified as “less.”

Questions regarding perceived difficulties in child-rearing were also divided into four scales as described for social support and then divided into two groups: “not at all” was classified as “no,” while the other responses were classified as “yes.”

### Statistical analysis

The chi-square test was used to assess the associations between social support and each sociodemographic characteristic and socioenvironmental condition related to child-rearing. For the statistical analysis, infant age was divided into two groups based on the median (27 months). Developmentally, 27 months is considered the stage at which an ego has developed, as well as the period in which mothers change their approaches to child-rearing. Logistic regression analysis was then conducted to identify predictive factors influencing social support. Analysis of the sociodemographic and socioenvironmental factors was performed using the chi-square test according to social support in each category. In all the analyses, *p* < 0.05 indicated statistical significance.

## Results

A total of 459 questionnaires were returned (response rate 46.2%), 388 of which were from mothers themselves and had been completed with regard to all of the questions regarding social support. There were no marked differences in the percentage of respondents between municipalities compared with the proportion of questionnaires sent within each municipality. As this study focused on socioenvironmental changes, the responses of 215 of 388 mothers living in houses different from those before the disaster were included in the analysis.

### Sociodemographic characteristics

The sociodemographic characteristics of mothers included in the statistical analysis are shown in Table 1. The mean age of their infants was 26.0 months (standard deviation [SD], 10.2). The range of infant age was 6 to 42 months, and age distribution of infants was 10.2% for age 0 (< 12 months), 29.3% for age 1 (< 24 months), 38.1% for age 2 (< 36 months), and 21.9% for age 3 (≤ 42 months). Two thirds of the mothers were employed, 32.6% were regularly employed, and 27.4% ware irregularly employed. More than half (54.9%) of the mothers lived in independent housing, while others lived in non-independent housing, such as multi-family apartments (30.2%) or public temporary housing (11.2%). In addition, most of the mothers were from nuclear family households (67.0%). More than half of the mothers (58.6%) had pre-disaster acquaintances living in the current residential area. Most of the mothers (86.0%) used at least one child support resource, such as nurseries, regional child support centers, and mothers’ self-help groups. However, 12.6% did not use any child support resources. More than half (56.3%) of the mothers reported having difficulties in child-rearing.

**Table 1:** Sociodemographic and socioenvironmental characteristics of mothers living in a house different from their house before the disaster (*n* = 215)

	Number	Percent
Sex of infant		
Male	113	52.6
Female	101	47.0
Unknown	1	0.5
Age of infant (range 6 to 42 months)		
≤ 26 months	105	48.8
≥ 27 months	109	50.7
Unknown	1	0.5
Mother’s age group		
20s	60	27.9
30s	134	62.3
40s	21	9.8
Employment status		
Unemployed	83	38.6
Irregular employment	59	27.4
Regular employment	70	32.6
Unknown	3	1.4
Housing		
Non-independent housing	96	44.7
Independent housing	118	54.9
Unknown	1	0.5
Household structure		
Nuclear family	144	67.0
Extended family	70	32.6
Unknown	1	0.5
Parent(s) living with infant		
Both (mother and father)	205	95.3
Only mother	8	3.7
Unknown	2	0.9
Order of target infant in family living together		
First child	119	55.3
Second child or more	96	44.7
Support from relatives not living together		
No	163	75.8
Yes	50	23.3
Unknown	2	0.9
Acquaintances in the neighborhood since before the disaster		
None	89	41.4
Yes	126	58.6
Use of child support resources		
None	27	12.6
At least one resource	185	86.0
Unknown	3	1.4
Perceived difficulties in child-rearing		
Yes	121	56.3
No	94	43.7
Social support score (mean/SD)		
Mental support (range 5–20)	16.0	3.3
Mental/physical place of comfort (range 5–20)	14.7	3.3
Child-rearing support (range 6–24)	17.9	4.5

With regard to the reliability of the social support in the child-rearing score, the Cronbach’s alpha of mental support was 0.788, with values of 0.712 for mental/physical place of comfort and 0.824 for child-rearing support. In addition, correlations among all of the social support categories were statistically significant. The coefficient of correlation was 0.308 (*p* < 0.001) between mental support and mental/physical place of comfort, 0.679 (*p* < 0.001) between mental support and child-rearing support, and 0.472 (*p* < 0.001) between mental/physical place of comfort and child-rearing support.

### Factors associated with social support

The chi-square test was used to assess factors associated with each of the social support categories (Table 2). Factors that were significantly associated with obtainment of mental support were having a female infant, having an extended household, and no perceived difficulties in child-rearing. Factors associated with mental/physical place of comfort were the presence of pre-disaster acquaintances, use of child support resources, and no perceived difficulties in child-rearing. Finally, factors associated with child-rearing support were regular employment, living in independent housing, having an extended household, obtaining support from relatives not living together, presence of pre-disaster acquaintances, and no perceived difficulties in child-rearing. However, there were no significant associations between infant age, mother’s age, parent(s) living with infant, birth order of target infant among siblings if any, and any social support categories. Furthermore, we investigated the relationship between infant age and social support score in each category and found that there was no significant correlation between them.

**Table 2:** Factors related to social support among mothers living in a house different from their house before the disaster (*n* = 215)

	Mental support					Mental/physical place of comfort					Child-rearing support				
	Few (*n* = 107)		Many (*n* = 108)		*p* value	Few (*n* = 87)		Many (*n* = 128)		*p* value	Few (*n* = 106)		Many (*n* = 109)		*p* value
	*n*	%	*n*	%		*n*	%	*n*	%		*n*	%	*n*	%	
Sex of infant															
Male	64	56.6	49	43.4	0.028	49	43.3	64	56.6	0.316	59	52.2	54	47.8	0.330
Female	42	41.6	59	58.4		37	36.6	64	63.4		46	45.5	55	54.5	
Age of infant (range 6 to 42 months)															
≤ 26 months	54	51.4	51	48.6	0.682	48	45.7	57	54.3	0.105	58	55.2	47	44.8	0.076
≥ 27 months	53	48.6	56	51.4		38	34.9	71	65.1		47	43.1	62	56.9	
Mother’s age group															
20s	26	43.3	34	56.7	0.501	25	41.7	35	58.3	0.958	28	46.7	32	53.3	0.214
30s	70	52.2	64	47.8		54	40.3	80	59.7		71	53.0	63	47.0	
40s	11	52.4	10	47.6		8	38.1	13	61.9		7	33.3	14	66.7	
Employment status															
Unemployed	48	57.8	35	42.2	0.115	34	41.0	49	59.0	0.950	54	65.1	29	34.9	0.001
Irregular employment	24	40.7	35	59.3		24	40.7	35	59.3		24	40.7	35	59.3	
Regular employment	33	47.1	37	52.9		27	38.6	43	61.4		26	37.1	44	62.9	
Housing															
Non-independent housing	51	53.1	45	46.9	0.343	43	44.8	53	55.2	0.215	58	60.4	38	39.6	0.003
Independent housing	55	46.6	63	53.4		43	36.4	75	63.6		47	39.8	71	60.2	
Household structure															
Nuclear household	79	54.9	65	45.1	0.041	61	42.4	83	57.6	0.350	84	58.3	60	41.7	< 0.001
Extended household	28	40.0	42	60.0		25	35.7	45	64.3		22	31.4	48	68.6	
Parent(s) living with infant															
Only mother	4	50.0	4	50.0	0.634^†^	3	37.5	5	62.5	0.597^†^	2	25.0	6	75.0	0.149^†^
Both (mother and father)	102	49.8	103	50.2		82	40.0	123	60.0		103	50.2	102	49.8	
Order of target infant in family living together															
First child	57	47.9	62	52.1	0.542	55	46.2	64	53.8	0.055	63	52.9	56	47.1	0.234
Second child or more	50	52.1	46	47.9		32	33.3	64	66.7		43	44.8	53	55.2	
Support from relatives not living together															
No	84	51.5	79	48.5	0.351	67	41.1	96	58.9	0.889	89	54.6	74	45.4	0.010
Yes	22	44.0	28	56.0		20	40.0	30	60.0		17	34.0	33	66.0	
Acquaintances in the neighborhood from before the disaster															
None	48	53.9	41	46.1	0.304	45	50.6	44	49.4	0.011	61	68.5	28	31.5	< 0.001
Yes	59	46.8	67	53.2		42	33.3	84	66.7		45	35.7	81	64.3	
Use of child support resources															
None	13	48.1	14	51.9	0.878	17	63.0	10	37.0	0.010	12	44.4	15	55.6	0.607
At least one resource	92	49.7	93	50.3		68	36.8	117	63.2		92	49.7	93	50.3	
Perceived difficulties in child-rearing															
Yes	69	57.0	52	43.0	0.015	61	50.4	60	49.6	0.001	74	61.2	47	38.8	< 0.001
No	38	40.4	56	59.6		26	27.7	68	72.3		32	34.0	62	66.0	

“Unknown” was excluded from analysis

Chi-square test or Fisher’s exact test (†) was conducted

Next, we performed logistic regression analysis according to each of the three categories of social support for child-rearing with all factors that were used in the chi-square test (Tables 3, 4, and 5). Factors significantly associated with mental support were infant sex and extended household, and factors associated with obtainment of a mental/physical place of comfort were the presence of pre-disaster acquaintances, use of child-support resources, and no perceived difficulties in child-rearing. The four factors that were significantly associated with obtainment of child-rearing support were extended household, obtaining support from relatives not living together, presence of acquaintances in the neighborhood from before the disaster, and no perceived difficulties in child-rearing.

**Table 3:** Factors related to the obtainment of social support by mental support category (*n* = 215)

	Mental support (forced entry) (*p* = 0.101)§			Mental support (stepwise backward elimination) (*p* = 0.005)§		
	AOR	95% CI	*p* value	AOR	95% CI	*p* value
Sex of infant						
Male	1.00			1.00		
Female	1.90	1.05–3.45	0.035	1.88	1.06–3.22	0.030
Age of infant (range 6 to 42 months)						
≤ 26 months	1.00					
≥ 27 months	1.09	0.60–1.99	0.783			
Mother’s age group						
20s	1.00					
30s	0.92	0.45–1.86	0.812			
40s	0.83	0.27–2.51	0.741			
Employment status						
Unemployed	1.00					
Irregular employment	1.86	0.84–4.11	0.124			
Regular employment	1.19	0.57–2.49	0.646			
Housing						
Non-independent housing	1.00					
Independent housing	1.10	0.55–2.19	0.790			
Household structure						
Nuclear household	1.00			1.00		
Extended household	2.16	0.97–4.83	0.060	2.31	1.22–4.37	0.010
Parent(s) living with infant						
Only mother	1.00					
Both (mother and father)	2.99	0.54–16.47	0.208			
Order of target infant in family living together						
First child	1.00					
Second child or more	0.69	0.35–1.35	0.273			
Support from relatives not living together						
No	1.00			1.00		
Yes	2.06	0.96–4.41	0.63	2.03	0.99–4.15	0.052
Acquaintances in the neighborhood from before the disaster						
None	1.00					
Yes	1.04	0.53–2.04	0.911			
Use of child support resources						
None	1.00					
At least one resource	0.99	0.40–2.48	0.986			
Perceived difficulties in child-rearing						
Yes	1.00					
No	1.60	0.86–2.95	0.136			

Logistic regression analysis was performed

All the factors shown in Table 2 were included in the analysis, namely, sex of infant, age of infant, mother’s age group, employment status, housing, household structure, parent(s) living with infant, order of target infant in family living together, support from relatives not living together, acquaintance in the neighborhood from before the disaster, use of child support resources, and perceived difficulties in child-rearing. “Unknown” was excluded from the analysis

*AOR* adjusted odds ratio, *CI* confidence interval,  § *p*-value for the model

**Table 4:** Factors related to obtainment of social support by mental/physical place of comfort category (*n* = 215)

	Mental/physical place of comfort (forced entry) (*p* = 0.011)§			Mental/physical place of comfort (stepwise backward elimination) (*p* < 0.001)§		
	AOR	95% CI	*p* value	AOR	95% CI	*p* value
Sex of infant						
Male	1.00					
Female	1.23	0.65–2.30	0.525			
Age of infant (range 6 to 42 months)						
≤ 26 months	1.00					
≥ 27 months	1.28	0.68–2.39	0.448			
Mother’s age group						
20s	1.00					
30s	1.19	0.57–2.47	0.651			
40s	0.98	0.31–3.14	0.973			
Employment status						
Unemployed	1.00					
Irregular employment	0.72	0.31–1.67	0.446			
Regular employment	0.72	0.33–1.57	0.414			
Housing						
Non-independent housing	1.00					
Independent housing	1.34	0.66–2.74	0.418			
Household structure						
Nuclear household	1.00					
Extended household	1.06	0.45–2.50	0.893			
Parent(s) living with infant						
Only mother	1.00					
Both (mother and father)	1.77	0.31–10.02	0.521			
Order of target infant in family living together						
First child	1.00					
Second child or more	1.24	0.62–2.49	0.549			
Support from relatives not living together						
No	1.00					
Yes	1.22	0.55–2.70	0.623			
Acquaintances in the neighborhood from before the disaster						
None	1.00			1.00		
Yes	1.79	0.90–3.58	0.097	1.88	1.03–3.44	0.040
Use of child support resources						
None	1.00			1.00		
At least one resource	4.15	1.59–10.80	0.004	3.96	1.61–9.75	0.003
Perceived difficulties in child-rearing						
Yes	1.00			1.00		
No	3.12	1.59–6.13	0.001	3.10	1.63–5.88	0.001

Logistic regression analysis was performed

All the factors shown in Table 2 were included in the analysis, namely, sex of infant, age of infant, mother’s age group, employment status, housing, household structure, parent(s) living with infant, order of target infant in family living together, support from relatives not living together, acquaintance in the neighborhood from before the disaster, use of child support resources, and perceived difficulties in child-rearing. “Unknown” was excluded from the analysis

*AOR* adjusted odds ratio, *CI* confidence interval,  § *p*-value for the model

**Table 5:** Factors related to the obtainment of social support by child-rearing support category (*n* = 215)

	Child-rearing support (forced entry) (*p* < 0.001)§			Child-rearing support (stepwise backward elimination) (*p* < 0.001)§		
	AOR	95% CI	*p* value	AOR	95% CI	*p* value
Sex of infant						
Male	1.00					
Female	1.08	0.55–2.12	0.830			
Age of infant (range 6 to 42 months)						
≤ 26 months	1.00			1.00		
≥ 27 months	1.96	0.98–3.91	0.057	1.79	0.93–3.43	0.079
Mother’s age group						
20s	1.00					
30s	1.08	0.49–2.36	0.852			
40s	2.00	0.55–7.26	0.292			
Employment status						
Unemployed	1.00					
Irregular employment	2.36	0.97–5.75	0.058			
Regular employment	2.40	1.04–5.53	0.040			
Housing						
Non-independent housing	1.00					
Independent housing	1.35	0.63–2.89	0.441			
Household structure						
Nuclear household	1.00			1.00		
Extended household	2.47	1.04–5.88	0.040	3.41	1.62–7.18	0.001
Parent(s) living with infant						
Only mother	1.00					
Both (mother and father)	1.54	0.24–9.74	0.647			
Order of target infant in family living together						
First child	1.00					
Second child or more	0.66	0.30–1.44	0.298			
Support from relatives not living together						
No	1.00			1.00		
Yes	4.86	2.02–11.68	< 0.001	5.30	2.29–12.27	< 0.001
Acquaintances in the neighborhood from before the disaster						
None	1.00			1.00		
Yes	2.70	1.28–5.69	0.009	2.84	1.46–5.52	0.002
Use of child support resources						
None	1.00					
At least one resource	0.57	0.20–1.61	0.285			
Perceived difficulties in child-rearing						
Yes	1.00			1.00		
No	3.57	1.74–7.33	0.001	3.32	1.70–6.49	< 0.001

Logistic regression analysis was performed

All the factors shown in Table 2 were included in the analysis, namely, sex of infant, age of infant, mother’s age group, employment status, housing, household structure, parent(s) living with infant, order of target infant in family living together, support from relatives not living together, acquaintance in the neighborhood from before the disaster, use of child support resources, and perceived difficulties in child-rearing. “Unknown” was excluded from the analysis

*AOR* adjusted odds ratio, *CI* confidence interval, § *p*-value for the model

Extended household was a factor among mothers who had obtained mental support and child-rearing support. The presence of pre-disaster acquaintances and perceived difficulties in child-rearing were common factors among mothers who had obtained social support in two categories, namely, mental/physical place of comfort and child-rearing support. The adjusted odds ratio (AOR) for the presence of pre-disaster acquaintances with mental/physical place of comfort was 1.88 (95% CI 1.03–3.44) and that for child-rearing support was 2.84 (95% CI 1.46–5.52). The AOR for no perceived difficulties in child-rearing was 3.10 (95% CI 1.63–5.88) for mental/physical place of comfort support, and it was 3.32 (95% CI 1.70–6.46) for child-rearing support.

## Discussion

Factors associated with social support among mothers who were living in a house different from that before the disaster were infant sex, living with extended family, obtaining support from relatives not living together, presence of pre-disaster acquaintances, use of child support resources, and no perceived difficulties in child-rearing.

The factor of living within an extended household was associated with the mental support category. The existence of family would enhance a sense of ease for mothers with the knowledge that some other member of the family can keep an eye on their children. Additionally, extended households were also associated with the child-rearing support category along with obtaining support from relatives not living together. The existence of family and relatives within their household or living nearby may be an important factor for mothers because it allows mothers to ask for help easily when needed [24].

Users of child support resources benefited more from having a mental/physical place of comfort than non-users of child-support resources. This result indicated that use of child support resources would contribute to increasing opportunities for mothers to meet someone with whom they can exchange talk about child-rearing and to comfortably allow the children to play [25].

Having no perceived difficulties in child-rearing was associated with mental/physical place of comfort support and child-rearing support but not with mental support. These results correspond to those of previous studies indicating associations between perceived difficulties in child-rearing and reduced levels of social support among mothers in the general population in Japan. Previous studies showed that factors associated with no perceived difficulties in child-rearing were having someone to talk to for advice and having someone who can take care of a child other than a child support center [26, 27]. The results of the present study in post-disaster communities were the same as those in previous studies in the general population; thus, mental/physical place of comfort support and child-rearing support are also key factors in post-disaster communities.

The presence of pre-disaster acquaintances was associated with mental/physical place of comfort support and child-rearing support. The presence of pre-disaster acquaintances was a socioenvironmental factor in the post-disaster community rather than a factor attributed to the mother or family. A previous study in the general population in Japan suggested that shallow relationships in the community could be a factor that influences a child’s caregiver to not obtain support [26]. This result could indicate that good relationships with people in the community have a positive influence on mothers with respect to child-rearing. The results of this study indicated that the presence of pre-disaster acquaintances had a positive role in social support among mothers with infants and preschool-aged children in a post-disaster community. A previous study among the general population 1 year after the Great East Japan Earthquake and Tsunami indicated that support from neighbors contributed to a reduction of psychological distress as well as the provision of support from family members in the area affected by the disaster [28]. Our study results also showed that the presence of pre-disaster acquaintances was positively associated with child-rearing social support, especially mental/physical place of comfort and child-rearing support. Pre-disaster acquaintances can be defined as people who mothers felt comfortable asking for help. This study area is characterized by robust ties within the community [29, 30]. Therefore, pre-disaster acquaintances would be key individuals likely to provide social support to mothers with infants and preschool-aged children under resource-poor conditions, such as the post-disaster setting. In addition, the presence of pre-disaster acquaintances could promote the establishment of helpful relationships in the redeveloping communities through the provision of social support. The presence of people who provide support to mothers with newborn babies would be helpful to maintain a healthy mental state with respect to child-rearing in post-disaster communities [31]. The results of this study suggested that the presence of pre-disaster acquaintances also contributes to the maintenance of mental health in mothers with infants and preschool-aged children when community ties have been negatively affected by a disaster.

Many changes in the physical environment of the study area, the Kesen region in Iwate Prefecture, have continued for several years since the disaster. For example, the construction of mounds of land has continued for more than 5 years even in the centers of communities. Access routes have changed several times because of road reconstruction and renovation, and many stores are still operating from temporary premises. Furthermore, people are only now, 6 years after the disaster, beginning to move from temporary accommodations to permanent housing [32–34]. These changes in the physical environment may affect socioenvironmental conditions, including social support, for the entire population living in areas affected by a disaster [35, 36], especially vulnerable populations, including mothers with infants and preschool-aged children. A study addressing resettlement and means of obtaining social support among residents of temporary housing in another prefecture affected by the same disaster indicated that people living close to previous community members were more likely to receive social support [37]. Perceived social support prior to Hurricane Katrina was reported to decrease the negative psychological effects of exposure to a natural disaster among low-income mothers [38]. Another study among older Taiwanese adults displaced by earthquakes showed that support by neighbors and social participation were significantly associated with lower levels of depressive symptoms 1 year after the earthquake [39]. Moreover, a previous study in a general population in Sweden indicated that having someone to talk to about personal problems was linked to reduced health problems, such as discouragement and poor self-rated health [40]. Thus, the presence of neighbors mitigates health problems and empowers parents even in general populations. Under fragile conditions, such as a post-disaster setting, the presence of pre-disaster acquaintances contributes to rich social support for mothers with infants and preschool-aged children following the significant changes in physical and social environments, including the breaking of community ties, associated with a disaster. Sharing these experiences with pre-disaster acquaintances may provide a sense of mutual aid.

Factors associated with obtaining social support in child-rearing among mothers were derived from the mothers themselves as well as family members, and also from socioenvironmental factors. These results suggested that it is necessary to consider both these factors when supporting for development process in post-disaster communities. The factors specifically needed to be considered were the family structure and perceived difficulties in child-rearing arising from the mother and family and the existence of pre-disaster acquaintances from the socioenvironmental factors. Consequently, our findings suggest that relationships with pre-disaster community members could encourage and empower mothers and families with infants and preschool-aged children in the reconstruction of the community in post-disaster residential areas.

This study had several limitations. First, the findings of this study cannot be generalized to all mothers living in post-disaster communities because this study was conducted in areas affected by a particular disaster, and mothers included in this study were not representative of those in other post-disaster communities. The study areas have specific characteristics, such as traditional community ties according to the main industries, including agriculture and fishing. In addition, mothers with perceived difficulties in child-rearing may not have completed the questionnaire in this study for several reasons, including being overly busy or not wishing to participate in the study.

Next, this study could not clarify the causal associations of social support with sociodemographic characteristics and socioenvironmental conditions due to the cross-sectional study design. Additionally, the measure of social support in this study could not be generalized because it was modified for adjustment within a post-disaster community, even though internal consistency was confirmed.

No statistically significant relationship was found between social support and infant age in this study. However, infant sex was statistically significant in the mental support category. These results need to be considered in future studies since infant age and sex are likely to affect child-rearing. Additionally, the educational level of the mother and economic status were not measured in this study based on the suggestion of local government officers who have a rich knowledge of the characteristics, values, and norms of the target communities and people. This was because mothers may feel uncomfortable in answering about their own educational status and economic status, possibly making it a deterrent to participation in this study. Moreover, the proportion of extended households of this study area was higher than that at the national level [41], and invisible economic activities may exist in the communities as mutual aid among neighbors [30]. Therefore, we presumed the association between economic status and obtaining social support by using variables of the housing and the household structure as an alternative indicator of economic status. From the result of this study, mothers of nuclear families obtained less social support compared with mothers of extended households. This condition possibly confounded with mother’s economic status because the expenditure of the nuclear families with small children could be higher than that of the family with small children living within an extended household.

There was a possibility that economic status and the ability of child-rearing based on the mother’s educational level could be confounded. Although we have not evaluated these associations in this study, such evaluations can be expected in future studies. We suggest inclusion of not only income but also the invisible economic activities within community-based relationships in the evaluation of economic status and also the health literacy and/or health-seeking skills of mothers by educational level especially with respect to child-rearing.

Additionally, information provided by the municipality offices of the study area indicated that approximately 16.5% of the target population had moved to the study area from other municipalities or regions. It is possible that mothers who reported the absence of pre-disaster acquaintances may have been relocated from other municipalities or regions. However, this study could not identify these new resident mothers in the disaster-affected communities. Therefore, it will be necessary to further examine the conditions of such new resident mothers, and these mothers should be included in the community network in future studies.

## Conclusions

Factors associated with the obtainment of social support in child-rearing among mothers in post-disaster communities were attributed not only to mothers themselves and family members but also to socioenvironmental factors such as the presence of pre-disaster acquaintances. The findings of this study indicated that the presence of pre-disaster acquaintances was one factor related to rich social support for mothers in child-rearing in post-disaster communities, especially with regard to mental/physical place of comfort support and child-rearing support.

During community reconstruction following a disaster, local government and support organizations should consider providing distinctive services to maintain relationships among community dwellers from before the disaster from the viewpoint of child-rearing support. It is necessary to take into account quantitative minorities and vulnerable populations during periods of long-term post-disaster community development as well as in the development of disaster preparedness plans for dealing with future disasters.

## Additional file

Additional file 1: Questions regarding social support in the present study by category. (DOCX 15 kb)
