# Supplementary material for: Factors associated with social support in child-rearing among mothers in post-disaster communities
Source: Environ Health Prev Med. 2018 Nov 7;23:58. doi: 10.1186/s12199-018-0747-7 (PMC6223082; doi:10.1186/s12199-018-0747-7)
Supplement: Supplementary file 1 — Questions regarding social support in the present study by category. (DOCX 15 kb) [file 12199_2018_747_MOESM1_ESM.docx]

Additional file 1. Questions regarding social support in the present study by category.

| Category | Definition for this study | Questions |
| --- | --- | --- |
| Mental support | Mental stability of the mother in childrearing through family support. | I raise my child/children alone.　*(reverse question)* |
|  |  | I can talk with my family about how the child/children’ was/were that day. |
|  |  | I can talk with my family about concerns regarding my child/children. |
|  |  | My family understands me well. |
|  |  | My family can help with childcare and housework on my behalf. |
| Mental/physical place of comfort | A place outside the home where the mother can talk about child-rearing and comfortably let children play. | I do not have the opportunity to let my child/children play with those of the same age group.  *(reverse question)* |
|  |  | I have a close fellow mother friend with whom we can trust each other’s children. |
|  |  | I do not have the opportunity to talk with parents with children of similar age as mine.*(reverse question)* |
|  |  | I am not familiar with families with children of similar age as mine. *(reverse question)* |
|  |  | I do not have the opportunity to talk about my child/children on a regular basis. |
| Child-rearing support | Professional/friendly give-and-take support in child-rearing. | I have someone I can talk to about childrearing (e.g. professionals such as doctors or health nurse) |
|  |  | There is someone near by to whom I can trust my child/children even for a short period of time |
|  |  | There is a close friend with whom I can talk about child-rearing. |
|  |  | I have someone to whom I can comfortably talk about my thoughts on childrearing |
|  |  | There is someone who can look after my child/children when I wish to go to the dentist/hairdressers etc. |
|  |  | There is someone I can talk to about my worries regarding childrearing |
